# A farmer-friendly tool for estimation of weights of pigs kept by smallholder farmers in Uganda

**DOI:** 10.1007/s11250-023-03561-z

**Published:** 2023-05-23

**Authors:** Karen Marshall, Jane Poole, Edwin Oyieng, Emily Ouma, Donald R. Kugonza

**Affiliations:** 1 International Livestock Research Institute, P.O Box 30709 - 00100, Nairobi, Kenya; 2 International Livestock Research Institute, c/o Bioversity International, P.O Box 24384, Kampala, Uganda; 3 School of Agricultural Sciences, College of Agricultural and Environmental Sciences, Makerere University, P.O Box 7062, Kampala, Uganda

**Keywords:** Smallholder, Pig, Uganda, Body weights, Heart girth, Weigh band

## Abstract

Pig keeping is important to the livelihoods of many rural Ugandans. Pigs are typically sold based on live weight or a carcass weight derived from this; however this weight is commonly estimated due to the lack of access to scales. Here, we explore the development of a weigh band for more accurate weight determination and potentially increased farmer bargaining power on sale price. Pig weights and varied body measurements (heart girth, height, and length) were collected on 764 pigs of different ages, sex, and breed types, from 157 smallholder pig keeping households in Central and Western Uganda. Mixed-effects linear regression analyses, with household as a random effect and the varied body measurements as a fixed effect, were performed to determine the best single predictor for cube root of weight (transformation of weight for normality), for 749 pigs ranging between 0 and 125 kg. The most predictive single body measurement was heart girth, where weight in kg = (0.4011 + heart girth in cm × 0.0381)^3^. This model was found to be most suitable for pigs between 5 and 110 kg, notably more accurate than farmers’ estimates, but still with somewhat broad confidence intervals (for example, ±11.5 kg for pigs with a predicted weight of 51.3 kg). We intend to pilot test a weigh band based on this model before deciding on whether it is suitable for wider scaling.

## Introduction

Within Uganda, pigs are being kept by an increasing number of smallholders in mixed crop-livestock systems. Pig enterprises provide a benefit to household members through income, often used for payment of school fees as well as unplanned expenses, provision of manure for cropping, and via adding diversity to the household livelihood portfolio (Babigumira et al., 2019; Ouma et al., 2014, 2015). Pigs are often preferred over other livestock species due to their relatively fast returns, being kept near the household (meaning they can be cared for by those who are homed based), limited land requirements, and because they can utilize household waste as feed. Currently the national pig herd is around 4.21 million (UBOS, 2020), a notable increase from a national herd size of 1.16 million in 1990 (FAOSTAT, 2023). This growth has primarily been in response to increased demand for pork, with Ugandans the highest per capital pork consumers in Eastern Africa (FAOSTAT, 2023). Whilst the sector offers increasing livelihood opportunities to women and men pig keepers as well as others in the pork value chain, such as input service providers, traders, and retailers, it also has some weaknesses.

One constraint to development of the Ugandan smallholder pig sector is around output markets and lack of transparency in trading. Output market constraints have been identified as lack of market information, limited marketing opportunities particularly in rural locations, and lack of capacity on pig live-weight estimation (Ouma et al., 2014). The latter is important as smallholders typically sell pigs from their farm gate to traders or butchers based on estimated liveweight or a carcase weight derived from this (weight is estimated due to lack of access to scales). It has been suggested buyers underestimates the weight of the animals in order to maximize on their margins, negatively affecting the producer’s revenue (Ouma et al., 2014).

A farmer-friendly tool that could assist in more accurate estimation of pig weight, thus giving farmers more bargaining power at time of sale, is a weigh band. Weigh bands are similar to tape-measures but display weight of animals alongside (or instead of) linear measurements. They are considered farmer friendly in that they are affordable and simple to use. Here, we explore the estimation of pig body weight from varied pig body measurements (heart girth, height, and length) with the view of producing a weigh band customized to the Uganda pig population. In addition to results of this analysis, summaries around pig-weight (such as weight for age) that could be derived from the same data are given, due to the scarcity of information to this end.

## Materials and methods

### Overview of data collection

Data was collected on 754 pigs of different ages, sex, and breed types, from 157 smallholder pig keeping households in four sites (Table 1). The sites were the districts of Masaka and Mpigi (neighboring districts) and Wakiso, in the Central regions of Uganda, Hoima in the Western region, and Kamuli in the Eastern region, all areas where there is a high importance of pigs to livelihoods of smallholders. Sites from different locations within Uganda were purposely selected to capture potential across-site-variation in pig weights and body measurements. Within each district, we liaised with the district veterinary officers to select two or three lower-level administration units (sub-counties, town councils, or divisions) with high pig populations and no current African Swine Fever outbreak or scare and from there collaborated with the sub-county veterinary or animal husbandry officers to generate a list of all pig keeping households within these. Households with two or more pigs were randomly selected from these lists. Participation was voluntary, and if a household did not wish to participate, a replacement household was randomly selected. Survey respondents, who were adult household members involved in the household pig enterprise, were 56% female and 44% male. Within each household all pigs were measured except for those that were noticeably pregnant (more than 10 weeks), overly aggressive, sick, or injured. Further only three piglets from any one litter were measured, selected at random. Data was collected between 29th November 2021 and 5th January 2022.

**Table 1:** Overview of data collected per site

Site as district	Sub-county, town council or city division	Number households	Number pigs	Range of number of pigs per household
Masaka & Mpigi	Nyendo Mukungwe division; Nkozi sub-county, Kammengo sub-county	43	191	2–8
Wakiso	Kyengera town council; Kasangati town council; Wakiso sub-county	38	174	2–10
Hoima	Kitoba sub-county; Kiziranfumbi sub-county; Busisi Division	38	195	3–10
Kamuli	Bugulumbya sub-county; Mbulamuti sub-county; Butansi sub-county	38	204	3–8
All		157	764	2–10

### Pig measurements and other collected data

Pig live weight was recorded, in kg and to one decimal place, by standing the pigs on a digital scale within a crate. Pig body measurements comprised heart girth, height, and length, all measured in cm to the closest 0.5 of a cm. Heart girth was measured as the circumference of the pig, immediately behind the front legs, using a measuring tape; height was measured from the rump (the highest point) to the ground, using a measuring stick; and length was measured as distance between the base of the ears to the base of the tail, using a measuring tape.

Other data collected included that on socio-demographic characteristics of the survey respondent and/or pig owner; perceived main breed of the pig, based on opinion of the enumerator from physical appearance, and then reported here as “local” or “exotic” when the main breed was perceived as the local (Ugandan breed) or an exotic breed, respectively; farmers estimated pig weight (asked before the pig was weighed or measured); pig sex (male or female), age (in months), castration status (if male), pregnancy status, and parity (if female); housing type as housed, tethered, and free-range; and pig body condition score (bcsbcsBCS) as 1, 2, 3, 4, and 5 for emaciated, thin, ideal, fat, and overfat, respectively. Photos were also taken. All data were collected using the ODK data collection tool (Hartung et al., 2010). Features of the dataset are presented using simple summary statistics.

### Summary of pig types

Table 2 summarizes pig numbers by sex, age, and perceived main breed. Overall animals, 46% were classified as females of the exotic breed, 30% as males of the exotic breed, 14% as females of the local breed, and 10% as males of the local breed. Pigs were from 0 to 60 months of age, with 33% of pigs between 3 and 6 months. Further information on the study pigs is provided in the “Results” section.

**Table 2:** Summary of numbers of pigs by age, sex and perceived main breed

Age (months)^a^	Female		Male		Total (% of total)
	Exotic	Local	Exotic	Local	
1	15	3	13	6	37 (5)
2	18	7	18	6	49 (6)
3	24	12	17	12	65 (9)
4	51	7	41	13	112 (15)
5	23	7	19	9	58 (8)
6	33	12	29	5	79 (10)
7	18	5	19	5	47 (6)
8	25	4	23	5	57 (7)
9	14	3	10	3	30 (4)
10	11	6	7	1	25 (3)
11	15	3	1	2	21 (3)
12	31	10	20	1	62 (8)
13–18	33	12	9	2	56 (7)
19–24	27	7	4	2	40 (5)
24–60	16	7	2	1	26 (3)
Total (% of total)	354 (46)	105 (14)	232 (30)	73 (10)	

^a^1 month refers to between 0 and 1 month of age, 2 months between more than one but less than 2 months of age, etc.

### Weight modelling

Mixed-effects linear regression analyses were performed to determine the best predictors (model) for weight, as well as the best single predictor, given a single predictor is amenable for use in a weigh band. As the model with weight as the response variable did not fit the assumptions of linear regression (i.e. linearity, independence, homoscedasticity, normality of residuals) weight was transformed: a cube root transformation was found to be the most appropriate. As there were only 15 animals between 125 and 205 kg, these were excluded, and the regression fitted for animals with weights ranging from 0 to 125 kg (which numbered 749). The random effect tested for inclusion in the model was the household, of which the final dataset had 157 (the number of pigs per household ranged from 2 to 10, with a mode of 4). Fixed effects tested for inclusion in the model are given in Table 3: via simple linear regression these were all significant predictors at *p*<0.20. The only variable excluded for testing in the final model due to having *p*>0.20 via simple linear regression was housing type.

**Table 3:** Independent variables tested as part of the regression modelling for weight, for the 749 animals with weights between 0 and 125 kg

Variable name (units)	Discrete variables: level, number in level Continuous variable: mean (standard deviation)
heart girth (cm)	70.2 (20.8)
height (cm)	82.8 (25.9)
length (cm)	50.2 (14.5)
age (months)	8.2 (6.9)
site	Hoima, 194; Kamuli, 202; Masaka and Mpigi, 183; Wakiso, 170
sex	Male, 302; female, 447
breed^1^	Exotic, 575; local, 174
Body condition score^2^	Thin, 52; normal, 529; fat, 179

^1^Classification on perceived main breed (many pigs likely to be crossbreed)

^2^Thin is body condition score (bcs) of 1 or 2; normal is bcs of 3; and fat is bcs of 4 or 5. This grouping was used due to few animals in the bcs 1 and 5 classes.

The regression was performed using the lmer function of the lme4 package of R (R Core Team, 2019). The best model was determined stepwise using the step function of the stats package of R (which performs backward elimination of random-effect terms followed by backward elimination of fixed-effect terms), with alpha values for the fixed and random effects of 0.10 and 0.05 respectively.

The final models were accessed for goodness considering the variance in weight explained by the model (i.e. the coefficient of (multiple) determination or *R*^2^ as well as accuracy of the regression estimates as the root mean square error (RMSE; equivalent to the standard deviation of the residuals) as well as the RMSE expressed as a percentage of the mean weight). Pseudo-*R*^2^s were generated using the r.squaredGLMM function of the MuMIn package of R. Residual plots (including standardized residuals versus predicted values and Q-Q residual plots) were also examined to check the assumptions of the model (as listed above). 95% confidence intervals were determined using the predictInterval function of merTools with 10,000 simulation samples of fixed effects.

### Ethical approvals

Approval for this study was obtained from the Uganda National Council for Science and Technology (UNCST, approval number A210ES); the International Livestock Research Institutes (ILRI’s) Institutional Research Ethics Committee, which is registered by the National Commission for Science, Technology and Innovation in Kenya (approval number ILRI-IREC2021-51); and ILRI’s Institutional Animal Care and Use Committee (ILRI-IACUC2021-27). Participation was voluntary, and informed consents were obtained from all respondents.

## Results and discussion

### Description of study pig population

The number of study pigs per household ranged between 2 and 10 (including piglets). Overall households, 42% of pigs were less than 5 months of age, 28% between 5 and 10 months (typical market-age), and 30% more than 10 months (those for marketing at a later age plus breeding animals). Pigs of breeding age were predominantly female (77%), with the remainder intact males (24%) or castrated males (9%, presumably still to be sold for slaughter). Considering breeding age females, 40%, 26%, 33%, and 3% of households had none, one, two or three, and four to seven, respectively. For intact boars, 79%, 18%, and 3% of households had none, one, or two, respectively.

Female pigs of breeding age were most commonly reported as parity one (38%) or parity two or three (20% and 13%, respectively), with few (4%) between parities four and eight. Twenty-five percent had not yet had a litter. The age range of first, second, and third parity animals was 10 to 19 months (with a mode of 12 months), 14 to 41 months (24 months), and 18 to 60 months (30 months), respectively. This implies a late age at first farrowing and long farrowing intervals in comparison to that achieved in more intensive systems (Rothschild, 1996; Chidgey et al., 2015; Koketsu et al., 2017).

Pig breed classification was based on the perceived main breed of an individual pig, many of whom would be crossbreeds (Babigumira et al., 2021), from physical appearance (due to the lack of pedigree recording). Overall, 77% of pigs were perceived to be mainly of an exotic breed and 23% mainly of the local or Ugandan breed (described in Babigumira et al., 2021). Exotic breed types were most commonly specified as Large White (56% of exotic pigs) and “Camborough” (used in reference to Camborough® from the Pig Improvement Company, 25%), with the remainder considered to comprise Landrace, Large Black, Duroc, Pietran, Saddleback, and unknowns. The proportion of pigs who were perceived to be mainly local was highest for Masaka (39%) followed by Hoima (33%), with fewer local pigs in Wakiso (13%) and Kamuli (6%).

On body condition, the majority of animals (69%) had a bcs of 3. Twenty-three percent of animals were considered too heavy (21% and 2% with bcs of 4 and 5, respectively), whilst 8% were considered too thin (7% and 1% with bcs of 2 and 1, respectively).

The majority (77%) of pigs were kept housed (in pig sties), whilst 22% were kept tethered and 2% free-ranged. The site of Hoima had less than half of the pigs housed (46%), whilst Kamuli and Masaka had most pigs housed (86% and 74%, respectively), and Wakiso had almost all pigs housed (99%). At a household level, 66% of households only housed their pigs, 17% only tethered their pigs, and 9% used a combination of housed and tethered, whilst the remaining 8% used free-ranging in combination with housing and/or tethering.

### Weights and body measurements

**Fig. 1:**
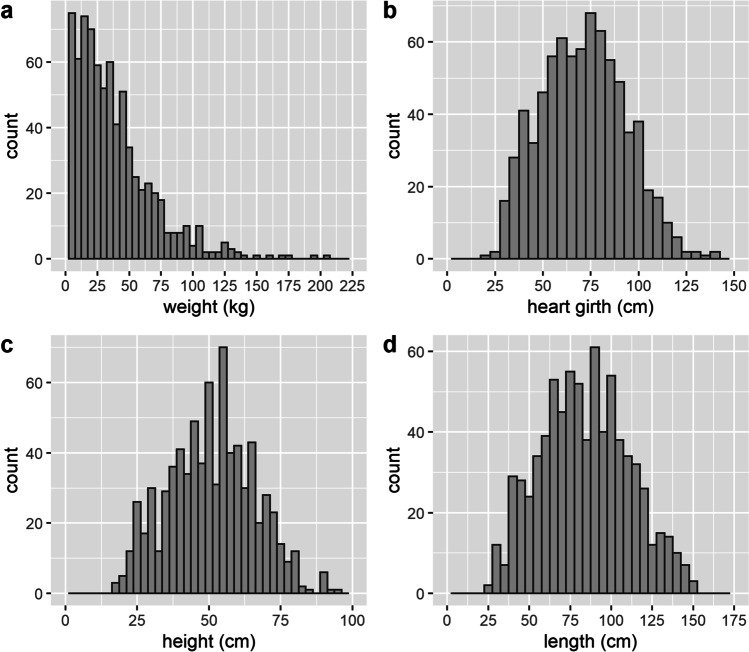
Distributions of pig weight (**a**), heart girth (**b**), height (**c**), and length (**d**)

**Fig. 2:**
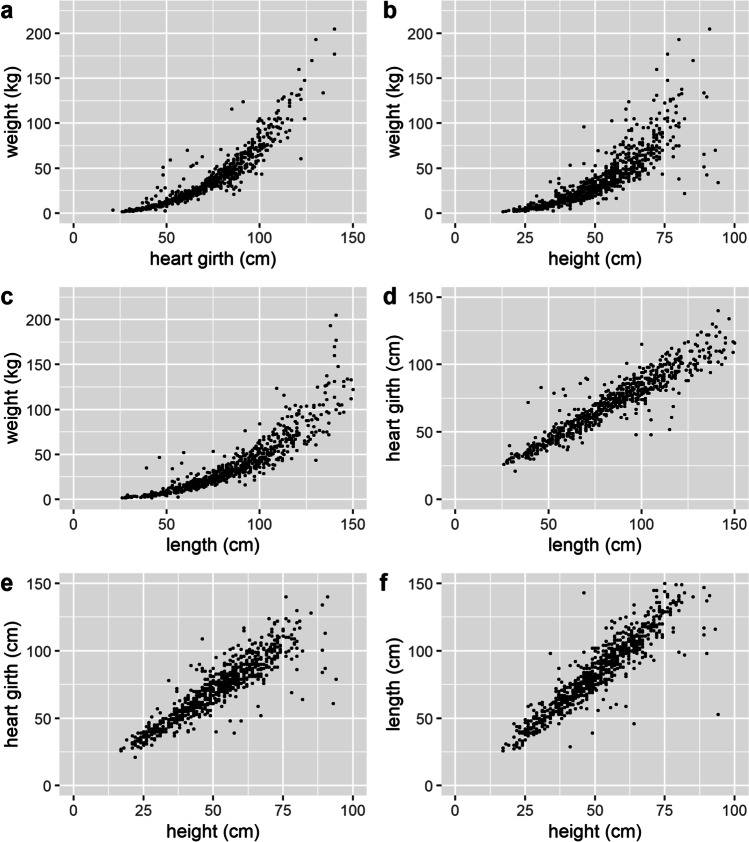
Scatterplots of weight and body measurements against each other: weight versus heart girth, height and length (**a**, **b**, **c**, respectively), heart girth versus length and height (**d**, **e**, respectively), and length versus height (**f**)

The distributions of weight and the varied body measurements (heart-girth, height, and length) are shown in Fig. 1. It is notable that whilst data for heart girth, height, and length were normally distributed, the weight data was right-skewed. Skewness of the weight data is due to the presence of more younger (and thus lower weight) animals in comparison to older (heavier) animals on the farms, arising from the sale of pigs from around 5 to 6 months onwards for slaughter. As shown in Fig. 2, the relationship between weight and the body measurements was non-linear, whereas those between the varied body measurements were linear.

**Fig. 3:**
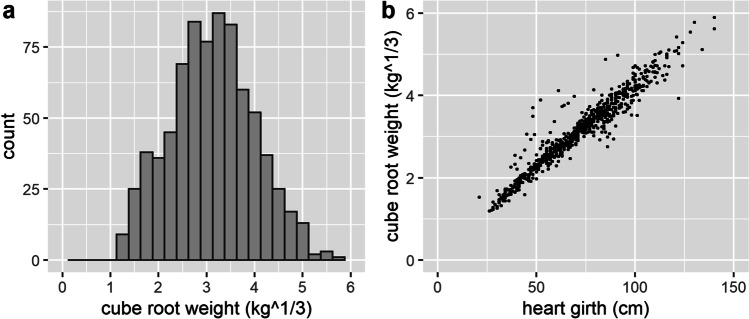
Distribution of cube root of weight (**a**) and scatterplot of cube root of weight and heart girth (**b**)

For the regression analysis, weight was cube root transformed for normality (Fig. 3). The correlations between the cube root of weight and the varied body measurements were strong, with coefficients of 0.96 for heart-girth, 0.95 for length, and 0.92 for height. The correlations between the varied body measurements were also all strong (with coefficients of 0.94 for heart girth and length, 0.92 for height and length, and 0.90 for heart girth and height, Fig. 2). These coefficients are similar to that previously reported (Groesbeck et al., 2002; Machebe and Ezekwe, 2010; Sungirai et al., 2014).

### Weights and body measurements by age

**Fig. 4:**
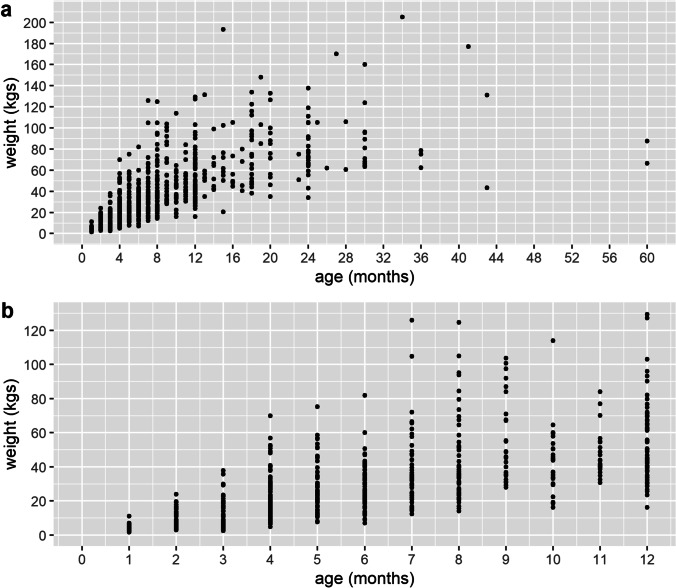
Weight versus age for (**a**) all animals and (**b**) animals to 12 months of age

Weights and body measurements for different age-ranges are given in Fig. 4 and Table 4. As sex was not significantly associated with weight (see “Modelling” section below) results are not sex disaggregated. At the ages of 6 months and 9 months, common market ages, the median weights were 27.0 kg and 46.0 kg, respectively. For fully grown pigs (here considering those 24 to 60 months), the median weight was 79.9 kg, and the medians of heart girth, height, and length were 100.5 cm, 71.5 cm, and 125.5 cm, respectively. The wide range of weights for a particular age is also notable. This is most likely mainly due to differences in management practices across households (Dione et al., 2014; Okello et al., 2021) but would also be due to other factors such as breed-type, natural variation, and age recall/estimation errors. Note that error in age assignment is suggested by pig numbers at half and full year intervals (6, 12, 18, months, etc.) being higher than what the general trend suggested, likely due to farmers estimating age to the closest half-year. Thus, these results should be interpreted with care.

**Table 4:** Summary of pig weights and body measurements for pig of different ages. Given is the number in each age group (N), medians (med), and first and third quartiles (Q1, Q3)

Age (months)^a^	*N*	Weight (kg)			Heart girth (cm)			Height (cm)			Length (cm)		
		Med	Q1	Q3	Med	Q1	Q3	Med	Q1	Q3	Med	Q1	Q3
1	37	3.6	2.7	5.0	34.0	30.0	37.0	24.0	22.0	26.0	37.0	31.0	42.0
2	49	7.6	5.3	12.6	44.0	39.0	52.0	32.0	28.0	38.0	52.0	46.0	62.0
3	65	11.6	7.0	17.3	49.0	41.0	57.0	36.0	30.0	41.0	58.0	47.0	66.0
4	112	20.4	13.7	28.4	58.0	52.0	69.3	43.0	37.8	49.0	70.0	61.8	81.0
5	58	25.1	19.1	34.6	66.5	59.3	75.0	48.0	42.0	53.9	77.0	68.3	85.3
6	79	27.0	19.9	35.6	70.0	60.0	77.0	50.0	44.5	55.3	79.0	71.0	90.0
7	47	34.3	24.7	45.7	76.0	68.5	86.0	54.0	46.0	60.0	87.0	75.5	99.0
8	57	34.5	26.2	51.5	76.0	67.0	86.5	54.0	49.0	60.0	90.0	79.0	102.0
9	30	46.0	37.5	67.4	84.0	78.3	94.8	61.0	56.0	69.8	100.5	93.3	113.0
10	25	37.0	30.2	50.7	81.0	71.0	83.0	55.0	49.0	63.0	94.0	78.0	104.0
11	21	43.6	39.7	54.5	84.0	80.0	93.0	57.0	54.0	61.0	97.0	91.0	100.0
12	62	49.1	39.5	69.0	86.5	75.0	95.0	61.0	56.3	69.8	103.5	92.3	116.8
13–18	56	58.8	48.8	77.3	90.0	83.8	100.0	65.3	60.8	72.3	113.0	100.0	124.3
19–24	40	75.5	63.0	96.5	99.5	93.0	106.0	67.2	62.8	74.5	116.5	108.0	134.0
24–60	26	79.9	65.2	105.8	100.5	92.8	117.5	71.5	68.0	75.3	125.5	116.3	135.8

^a^1 month refers to between 0 and 1 month of age, 2 months between more than one but less than 2 months of age, etc.

A similar study was performed on smallholder pigs in Western Kenya (Mutua et al., 2010) who measured weight, heart girth, and length and reported by age groups of ≤ 5 months (representing young animals), >5 and <10 months (market age), and ≥10 months (breeding age). When considering these age groups on our data and for weight, the means (standard deviations) were 17.4 (13.1), 38.2 (21.7), and 65.5 (31.8) kg, respectively. The latter (from our data) are higher (by 45%, 27%, and 64%, respectively) than that reported in the Kenyan study of 12 (6.1), 30 (11.4), and 42 (17.0) kg, respectively. Heart girth and length were also higher in this study compared by the Kenya one, though not as noticeably (by 6 to 13% for heart girth and 10 to 19% for length). This may reflect differences in breed compositions between the two studies (which were undertaken 10 years apart: increased use of exotic breeds over time has occurred in both Kenya and Uganda) and/or differences in management practices. The Kenyan study also reported sex of the pigs not to be associated with weight, as was found here.

### Weight modelling

Given the desire to predict weight from a single predictor, we modelled cube root of weight using heart girth, height or length as a single fixed effect, and household as a random effect. The final (reduced) models remained as these (Table 5), as household was significant as a random effect. Of the three models, cube root of weight ~ heart girth + household was the most predictive, with an *R*^2^ of 0.93 and RMSE expressed as a % of the mean cube root of weight of 6.8%. Household explained 19% of the variation after accounting for the fixed effects. Numerous other studies, which usually model weight rather than the cube root of weight, have also reported heart girth to be the highly predictive of weight (Groesbeck et al., 2002; Iwasawa et al., 2004; Machebe and Ezekwe, 2010; Mutua et al., 2010.; Paras et al., 2015; Panda et al., 2021).

**Table 5:** Modelling of cube root of weight. RMSE is the residual mean square error

Model	Coefficients (standard error)	Adjusted *R*^2^	RMSE	RMSE as a % of mean cube root of weight
heart girth + household	intercept 0.4011 (0.0334) heart girth 0.0381 (0.0004)	0.93	0.21	6.8
height + household	intercept 0.4723 (0.0481) height 0.0520 (0.0009)	0.86	0.29	9.5
length + household	intercept 0.5480 (0.0342) length 0.0306 (0.0004)	0.92	0.22	7.1
heart girth + height + length + site + breed + bcs + age + household	intercept: 0.4572 (0.0393) heart girth: 0.0180 (0.0010) height: 0.0088 (0.0012) length: 0.0114 (0.0008) breed local: −0.0443 (0.0187) bcs thin: −0.1832 (0.0324) bcs normal: −0.0607 (0.0194) age: 0.0048 (0.0015)	0.96	0.16	5.1

**Fig. 5:**
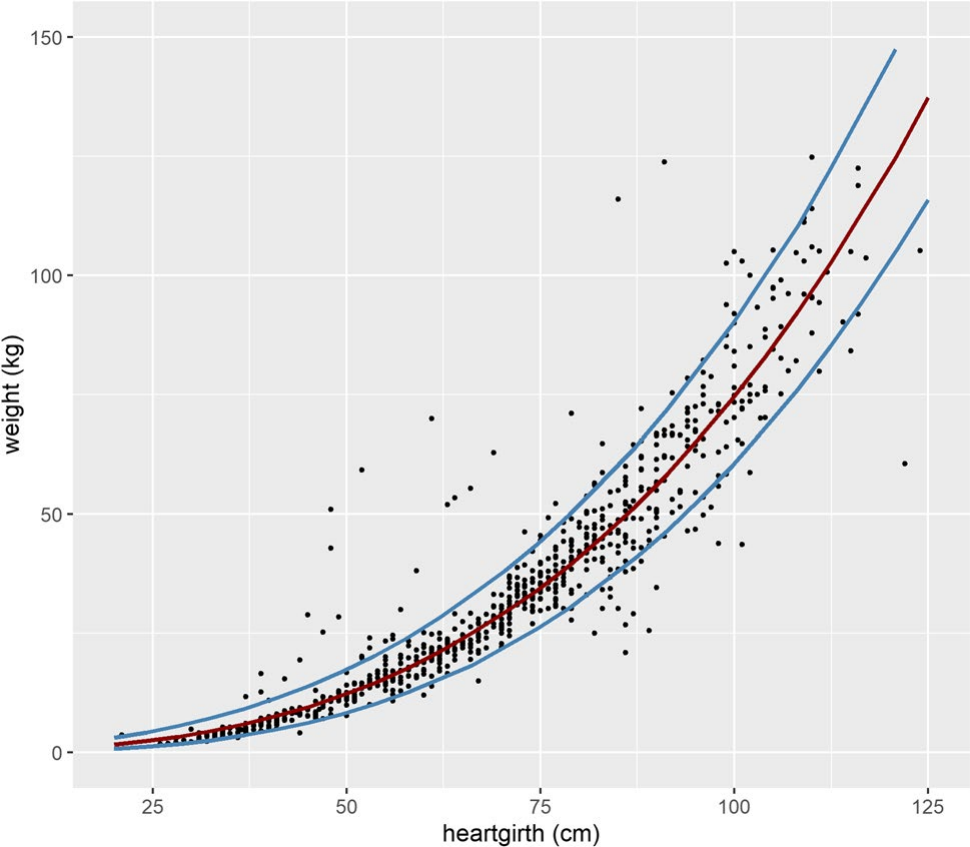
Predicted weight (red line) and 95% confidence intervals (blue line), for weight predicted from heart girth as weight in kg *=* (0.4011 *+* 0.0381 × heart girth in cm)^3^. The scatterplot shows all animals included in the dataset that the prediction was based on*.*

Predicting weight from heart girth as weight in kg = (0.4011 + heart girth in cm × 0.0381)^3^ (as per Table 5 but back-transforming from the cube root transformation) was explored further (note that whilst the random effect of household was included in the model as this gives the most appropriate fixed effect coefficients, it is not included in the prediction equation). A plot of predicted weight and the 95% confidence intervals are shown in Fig. 5, along with a plot of weight versus heart girth for the dataset the prediction equation was derived from. The 95% confidence intervals increase as predicted weight increases. For example, the 95% confidence intervals for animals with predicted weights of 4.4, 51.3, and 102.7 kgs were 2.5 to 7.1 kg (i.e. ± 2.3 kg), 40.9 to 63.9 kg (± 11.5 kg), and 85.2 to 122.5 (± 18.7 kg), respectively. Table 6 gives, for different actual weight ranges, summaries of residuals. The means of absolute residuals expressed as a % of the mean actual weights (mean relative residuals) were particularly high for animals less than 5 kg (at 48%) and 110 kg or more (at 24%) indicating the prediction is less accurate for these extreme weight ranges in comparison to the intermediate weight ranges (where the mean relative residuals ranged from 11 to 18%). For animals from 5 kg to less than 110 kg, the percentage of animals whose weight was predicted within 10% of their actual weight ranged from 31 to 59% (depending on the weight range) whilst between 62 and 93% of animals had their weight predicted within 20% of their actual weight. Considering animals between 50.0 and 59.9 kg (a typical market weight), the mean absolute residual was 9.5 kg corresponding to a mean relative residual of 17%, with predicted weights within 10% and 20% of actual weights for 31% and 70% of the pigs, respectively. For animals less than 50 kg, the predicted weights were, on average, overestimated, whilst for animals over 50 kg, they were underestimated.

**Table 6:** Comparisons of residuals between two models for predicting weight, for animals of different actual weight ranges

Weight range (kg)	Number of pigs	Mean actual weight (kg)	Heart-girth model				Multiple predictor model			
			Mean predicted weight (kg)	Mean residuals (kg)	Mean absolute residuals (kg)	Mean relative residual^a^ (%)	Mean predicted weight (kg)	Mean residuals (kg)	Mean absolute residuals (kg)	Mean relative residual^a^ (%)
0–4.9	45	3.5	4.8	−1.4	1.5	48	4.2	−0.7	0.9	13
5–9.9	65	6.9	8.0	−1.1	1.2	18	7.5	−0.5	0.9	13
10–19.9	135	14.9	15.5	−0.6	2.0	14	15.4	−0.5	1.9	13
20–29.9	127	24.3	24.7	−0.4	3.8	15	24.3	−0.1	3.2	11
30–39.9	107	34.4	35.4	−1.0	4.2	12	35.0	−0.6	3.9	13
40–49.9	88	44.6	45.3	−0.7	5.8	13	45.4	−0.8	5.8	14
50–59.9	54	54.4	51.0	3.4	9.5	17	52.1	2.2	7.4	12
60–69.9	41	64.9	61.0	3.9	9.8	15	64.9	0.0	7.9	10
70–79.9	35	74.1	72.2	1.8	7.9	11	73.7	0.4	7.2	6
80–89.9	15	84.7	83.7	1.1	9.0	11	84.2	0.5	5.5	11
90–99.9	16	94.4	88.6	5.8	11.4	12	89.9	4.4	10.6	11
100–109.9	13	103.8	94.7	9.1	16.1	16	95.4	8.4	11.9	18
110–125	8	117.9	89.1	28.8	28.8	24	97.3	20.6	21.5	12
0–125	749	35.5	34.9	0.6	5.1	16	35.2	0.3	4.3	13
5–109.9	696	36.6	36.2	0.4	5.1	14	36.5	0.1	4.3	12

^a^Relative residual for each animal were determined as the absolute residual for that animal expressed as a % of the animal’s weight

For comparison purposes, we also modelled cube root of weight using all terms in Table 3 plus the interaction of bcs with heart girth as fixed effects and household as a random effect. The final (reduced) model was cube root of weight ~ heart girth + height + length + breed + bcs + age + household. This model had an *R*^2^ of 0.96 and an RMSE expressed as a % if the mean cube root of weight of 5.1%, both slightly more favourable that than for the model based on heart girth only as discussed above. Coefficients for the discrete variables of breed and bcs were as expected: higher for exotic compared to the local breed and higher for a bcs of fat versus normal versus thin (Table 5). Terms removed from the model comprised site, sex, and the interaction of bcs with heart girth. The mean relative residuals for the different weight ranges (Table 6) ranged from 6 to 18% and were generally better than that for the heart girth only model and notably better for animals less than 5 kg (where the relative residual was 13% for this model versus 48% for the heart girth model) and more than 110 kg (12% versus 24%). For animals more than 5 and less than 100 kg, the proportion of animals whose weight was predicted within 10% of their actual weight ranged from 53 to 73% (versus 31% to 59% for the heart girth only model, Table 7), whilst those predicted within 20% of their actual weight ranged from 77 to 100% (versus 62% and 93%). For animals between 50 and 59.9 kg, the mean absolute residual and mean relative residual was 7.4 kg and 12%, respectively (in comparison to 9.51 kg and 17% for the heart girth only model), with predicted weights within 10% and 20% of actual weights for 54% and 80% of the pigs, respectively (in comparison to 31% and 70% for the heart girth only model). Predicted weights were over- and under-estimated following the same pattern as the heart girth only model.

**Table 7:** Comparisons between two models for predicting weight of the proportion of animals whose weight was estimated within 10% and 20% of their actual weight, for animals of different weight ranges

Weight range (kg)	% Animals with a predicted weight within the given % of their actual weight			
	Heart-girth model		Multiple predictor model	
	10%	20%	10%	20%
0–4.9	7	13	20	51
5–9.9	34	62	43	78
10–19.9	56	79	55	77
20–29.9	56	76	53	83
30–39.9	59	85	63	83
40–49.9	55	80	61	82
50–59.9	31	70	54	80
60–69.9	49	88	61	85
70–79.9	54	89	66	91
80–89.9	47	93	73	100
90–99.9	31	88	56	88
100–109.9	38	62	38	92
110–125	25	63	63	75
0–125	48	74	54	80
5–109.9	51	78	56	82

As expected, the model with the multiple predictors resulted in more accurate predictions than the heart girth only model, particularly for animals of less than 5 kg and 110 kg or more.

The models are that based on heart girth only, as weight in kg = (0.4011 + 0.0381 × heart girth in cm)^3^ (heart-girth model), and that based on multiple predictors, as weight in kg = (0.4572 + 0.0180 × heart girth in cm + 0.0088 × height in cm + 0.0114 × length in cm + −0.443 if local breed + −0.1832 if bcs thin + −00.0607 if bcs normal + 0.0048 × age in months)^3^ (multiple predictor model)

### Farmers estimated pig weight

**Fig. 6:**
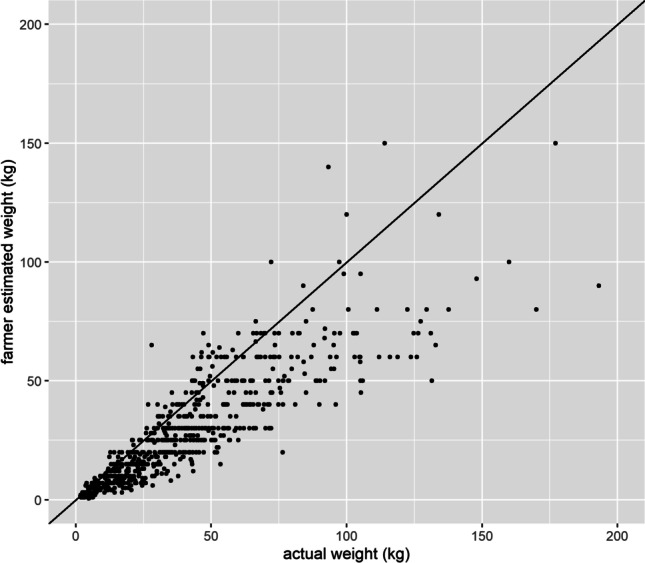
Farmer estimated weights versus actual weights of the same pigs

Farmers were asked to estimate the weights of their animals prior to the actual weights being taken. A comparison of farmer’s estimated versus actual weight is given in Fig. 6. Farmers mostly underestimated weight (83% of cases) but could also overestimated (15%) or estimate exactly (2%, mostly lower weight animals). Overall weight was underestimated, and more so for the heavier animals. For example, for pigs of 10 to 19.9 kgs, 50 to 59.9 kg (typical market weights), and 100 to 109.9 kg, farmers underestimated weight by, on average, 4.7 kg (equal to 24% of the mean weight), 15.5 kg (26%), and 35.5 kg (32%).

In comparing weight predicted from the heart girth model (as described above) to farmers estimated pig weight, the heart girth prediction is considerably more accurate. Considering animals between 5 and 109.9 kg, the mean relative residuals for the weight ranges shown in Table 6 ranged from 27 to 37% for farmer estimation versus 11 to 18% for the heart girth prediction. Further, the % of animals whose weight was estimated by farmers to within 10% of their actual weight was 8 to 21% in comparison to 31 to 59% for the heart girth model, whilst the % of animals whose weight was estimated by farmers to within 20% of their actual weight was 8 to 40% in comparison to 62 to 93% for the heart girth model.

### Comparison to previous studies

For the Western Kenya study of Mutua et al. (2010) predictions, equations were reported across all study animals as well as for sub-sets of animals based on age (up to 5 months, more than 5 and less than 10 months, and greater than 10 months). A mixed model approach was used with village fitted as the random effect and found to be significant. Final prediction equations were based on heart girth and length, with these variables explaining 88 to 91% of the variation in weight. Another study (Walugembe et al., 2014) used data from pigs located in the Kamuli district of Uganda (one of the four sites included in this study). Only fixed effects were fitted. They reported a single prediction equation for all animals, as well as for sub-sets of animals based on weight (less than 40 kg and 40 kg or more). The prediction equations were based on heart girth and body length for pigs less than 40 kg (explaining 89% of the variation in weight); heart girth, body length, height, and body width for pigs more than 40 kg (92%); and body length, heart girth, and body width for all animals (95%). They also reported heart girth to be the most important single predictor (though the prediction equation based on heart girth alone was not given). These models have a similar *R*^2^s to that presented here. A notable difference between this study and the previous studies (Mutua et al., 2010; Walugembe et al., 2014) is that non-normality is weight was handled by transformation in this study and by subsetting data based on either age or weight in the other studies. The advantage of the transformation approach taken here is that it results in a single prediction equation across all animals, which is more amenable to weigh band development.

In addition to the studies described here, numerous other studies have been undertaken on the relationship between pig weights and body measurements (Al Ard Khanji et al., 2018; Groesbeck et al., 2002; Kumari et al., 2020; Iwasawa et al., 2004; Machebe et al., 2010; Murillo and Valdez, 2004; Panda et al., 2021; Paras and Cu-Cordoves, 2015; Sungirai et al., 2014). However, these were conducted outside of East Africa ánd are thus less relevant due to differences in management strategies, particularly feeding regimes, as well as breed types (Ugandan pigs are typically crosses, including of the local “Ugandan” breed).

### Recommendations

The focus of this study was to predict a wide range of weights of pigs using a single predictor, such that a weigh band could be developed. The best single predictor was heart girth, which predicted weight with notably higher accuracy than farmer estimates but still relatively wide confidence intervals (for example, ±11.5 kg for pigs with a predicted weight of 51.3 kg). Besides accuracy of the prediction equation, utility of a weigh band depends on the user’s ability to correctly measure the heart girth, read the corresponding estimated weight, and understand that the real weight can be within a range around this estimate. To this end, training is recommended, not only of the women and men pig keepers, but also of other stakeholders including pig traders and extension officers. Moving forward, we plan to pilot test a weigh band based on the heart girth model presented here, to obtain user feedback as well as ascertain whether such a tool improves farmer bargaining power at time of pig sale. This pilot will consider both the accuracy of the tool and the perceptions, of pig keepers, traders, and other actors, on its use. Results of this pilot will determine whether the tool is deployed at larger scale or whether alternative approaches (such as an app based on the full model or increasing access to actual weight scales) are required. If the tool is deployed, it will be important that it is continually validated over time, due to the ongoing change in smallholder pig production systems in Uganda, including on breed type and management practices.

Looking towards the future, strong consideration should also be given to the use of image analysis for pig weight prediction (Fernandes et al., 2019 and 2020), particularly as the enabling environment for use of this technology (such as farmer utilization of smart phones and the Internet) improves.

## Data Availability

The dataset used in this study is available through https://hdl.handle.net/20.500.11766.1/FK2/IWXZQH
